# Bone mineral density, vitamin D receptor (VDR) gene polymorphisms, fracture risk assessment (FRAX), and trabecular bone score (TBS) in rheumatoid arthritis patients: connecting pieces of the puzzle

**DOI:** 10.1007/s10067-022-06048-8

**Published:** 2022-01-19

**Authors:** Manar R. Senosi, Hanan M. Fathi, Noha M. Abdel Baki, Othman Zaki, Ahmed M. Magdy, Tamer A. Gheita

**Affiliations:** 1 Rheumatology and Rehabilitation Department, Faculty of Medicine, Fayoum University, Fayoum, Egypt; 2 Rheumatology and Clinical Immunology Department, Faculty of Medicine, Cairo University, Cairo, Egypt; 3 Clinical Pathology Department, Faculty of Medicine, Damietta University, Damietta, Egypt; 4 Radio-Diagnosis Department, Faculty of Medicine, Fayoum University, Fayoum, Egypt

**Keywords:** Bone mineral density, DAS28, FRAX, Rheumatoid arthritis, TBS, VDR

## Abstract

**Purpose:**

To assess vitamin D receptor (VDR) gene polymorphisms and bone mineral density and to investigate the possible risk factors of osteoporosis and fracture in rheumatoid arthritis (RA).

**Methods:**

A total of 97 RA patients and 45 matched controls were enrolled. Serum vitamin D level, VDR genotyping, dual-energy X-ray absorptiometry (DEXA) scan, trabecular bone score (TBS), and fracture risk assessment (FRAX) in 10 years were assessed. Disease activity score (DAS28) and modified health assessment questionnaire (MHAQ) were measured.

**Results:**

The mean age of the patients was 47.9 ± 8.9 years; 85 females, 12 males (F:M 7.1:1) and mean disease duration 9.4 ± 6.2 years. DAS28 was 4.52 ± 1.04 and MHAQ 0.6 ± 0.4. There was a significant difference between cases and controls as regards DEXA and FRAX (*p* < 0.0001) but the TBS and VDR genotyping were comparable (*p* = 0.29 and *p* = 0.12, respectively). The vitamin D level was comparable with the control (9.3 ± 6.5 vs 10.4 ± 7.5 ng/mL, *p* = 0.4). None of the patients was receiving anti-osteoporotic therapy or biologic therapy. There was a significant association between the presence of osteoporosis and age, disease duration, menopause, and rheumatoid factor (RF) positivity. The TBS was significantly lower and FRAX higher in patients with positive RF and anti-CCP. FRAX was significantly related and the TBS inversely with the age, disease duration, serum uric acid, alkaline phosphatase, and MHAQ.

**Conclusions:**

Reduced BMD and increased tendency to fractures are remarkable in RA patients. Vitamin D level was decreased in patients and control, and VDR gene polymorphisms were not linked to RA. TBS and FRAX are effective tools to assess osteoporotic fractures in RA.

**Table:** 

**Key Points** *• Reduced bone mineral density (BMD) and increased tendency to fractures are remarkable in rheumatoid arthritis (RA) patients.* *• Vitamin D level was decreased in patients and control, and VDR gene polymorphisms were not linked to RA.* *• Trabecular bone score (TBS) and fracture risk assessment (FRAX) in 10 years are effective tools to assess osteoporotic fractures in RA.*

## Introduction

Rheumatoid arthritis (RA) is a chronic systemic autoimmune disease, with variable clinical expression. It arises more frequently in females than in males, being predominantly observed in the elderly. It can be ranging from mild self-limiting disorder to a very aggressive form, resulting in joint destruction and progressive disability [1]. Bone is a dynamic tissue that is continuously renewed throughout life by the process of bone remodeling. There is a disturbance in this equilibrium with increased resorption by osteoclasts and less bone formation by osteoblasts, leading bone to become weaker and more prone to fractures [2].

The remodeling process is the result of interactions between these cells (osteoclasts and osteoblasts) and multiple molecular agents, including growth factors, hormones, and cytokines [3]. Altered bone remodeling, whether excessive resorption and/or impaired formation, is a key risk for osteoporotic fracture [4, 5]. The quality of life in RA patients has been markedly affected by osteoporosis and related fragility fractures, being one of the most common complications [6]. Multi-factorial conditions have been involved in RA development, but genetic factors are considered to be strong determinants of these conditions. One of these influencing genes in RA progress is the vitamin D receptor (VDR) gene and its polymorphisms [7]. There are several polymorphic sites in the 3′ region of the VDR human gene identified by restriction endonuclease enzymes TaqI, BsmI, ApaI, and another variant in the exon 2 recognized by FokI. These polymorphisms may affect the response to various dietary components with the potential raised risk for development of pathology, being demonstrated on a large scope by functional involvement of the alleles of the VDR on calcium homeostasis and bone mineralization [8].

The fracture risk assessment (FRAX) in 10 years tool was developed to evaluate the 10-year risk of bone fragility fractures in females and males older than 40 years. This tool relies on selected clinical risk factors for fractures, where bone mineral density (BMD) was measured at the femoral neck. Adjusted versions were established, according to the local epidemiological data, and its availability for most countries in the world [9]. Osteoporosis is diagnosed using dual-energy X-ray absorptiometry (DXA) which measures BMD [10], but one of its limitations is its low value as an independent predictor of fracture risk [11, 12]. To overcome this limitation, the trabecular bone score (TBS) was developed to determine the bone’s micro-architecture [13]. Thus, the objective of the current work was to assess VDR gene polymorphisms and BMD in Egyptian RA patients and to investigate the possible risk factors of osteoporosis and of fracture.

## Patients and methods

The study included 97 RA patients fulfilling the American College of Rheumatology/European League Against Rheumatism (ACR/EULAR) classification criteria [14] and 45 age and sex matched healthy controls. Any patient with metabolic disorders, and/or secondary causes of osteoporosis as hyperthyroidism, premature menopause (< 45 years), chronic liver disease, or diabetes mellitus, was excluded. The patients’ consents were obtained and the study was approved by the local ethics committee, in accordance with the 1964 Helsinki Declaration.

Patients were subjected to full history taking including menstrual and menopausal age, diet, sun exposure, smoking, current medications, prior fractures, family history of hip fracture as well as clinical examination, and assessment of the body mass index (BMI). Visual analog scale (VAS)—pain [15], modified health assessment questionnaire (MHAQ) [16], and disease activity score (DAS28) [17] were assessed. The presence of metabolic syndrome (MetS) was considered [18].

Laboratory investigations included complete blood picture, erythrocyte sedimentation rate, C-reactive protein, liver and kidney functions, thyroid stimulating hormone (TSH), parathormone (PTH), alkaline phosphatase (ALP), glycated hemoglobin (HbA1c), lipid profile, rheumatoid factor, anti-citrullinated cyclic peptide, serum calcium, and 25-hydroxy vitamin D.

### Vitamin D receptor gene genotyping

A total of 2 mL of fresh whole blood was collected in EDTA-containing tubes and stored in − 80 °C. The genomic DNA was isolated using the isolation kit (Qiagen, Germany). DNA samples were stored at − 20 °C; DNA concentration was determined using a NanoDrop lite Spectrophotometer (Thermo Scientific). Genotyping of Fok-I (rs2228570) and Taq-I (rs731236) was performed using TaqMan SNP Genotyping Assay. Real-time polymerase chain reaction (PCR) was performed using 5 μL TaqMan Genotyping Master Mix (2 ×), 0.5 μL TaqMan SNP Genotyping Assay (TaqMan probes) (20 ×), 3.5 μL DNase Free Water, and 1 μL DNA (50 ng), to bring the final reaction volume to 15 μL. PCR consisted of a hot starts at 95 °C for 10 min followed by 40 cycles of 94 °C for 15 s and 60 °C for 1 min. All assays were performed using TaqMan Genotyping Master Mix on 96-well plates.

The BMD was assessed using the DXA scan (Lunar Prodigy, Madison, WI, USA) at the lumbar spine L1–L4, left femoral neck, and forearm. BMD measurement at or below − 2.5 SD from the optimal peak bone density of healthy young adult of the same sex was considered osteoporotic [10]. The patient with the most reduced BMD in the spine, femur, or forearm was categorized as having osteopenia or osteoporosis accordingly. TBS is a DXA-derived parameter based on the gray-level analysis that measures the microarchitecture or bone tissue distribution. TBS is good if the distribution of spaces is uniform. Lumbar spine (LS) TBS *T*-scores were calculated through the TBS iNsight software (Medimaps group, France) version 3.0 and were performed on the same vertebrae as those used for the BMD measurements.

The FRAX was computed using the algorithm available online at http://www.shef.ac.uk/FRAX (calculating the mean Jordanian and Turkish tools) with 9 clinical variables: age, BMI, previous fracture, hip fracture in a parent, alcohol use, smoking status, systemic glucocorticoids, RA, secondary osteoporosis, and femoral neck BMD.

### Statistical analysis

Data was collected and analyzed using the Statistical Package for the Social Sciences (SPSS) software version 22. Data was presented as numbers and percentages or mean and standard deviation. Normality of distribution was considered using the one-sample Kolmogorov–Smirnov test. For comparisons, the independent samples *t*-test, chi-square test, and one-way ANOVA test were used. The Spearman correlation test was considered to test the association between quantitative variables. Regression analysis was conducted to determine the independent risk factors predicting osteoporosis. ROC curve was analyzed for VDR gene expression as a predictor for RA. The *p*-value < 0.05 was considered significant.

## Results

**Fig. 1:**
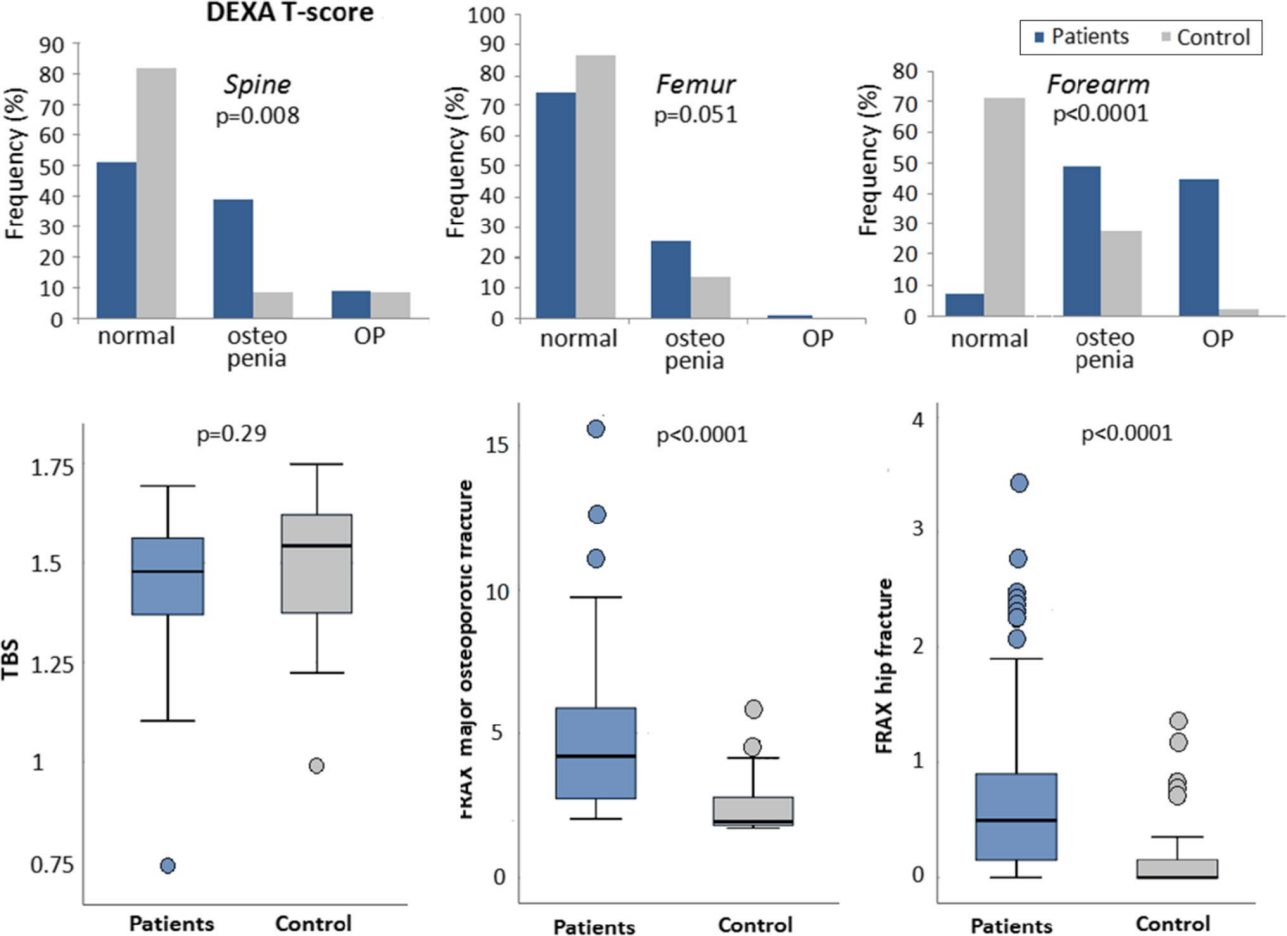
Dual-energy X-ray absorptiometry (DEXA), trabecular bone score (TBS), and fracture risk assessment (FRAX) in 10 years in rheumatoid arthritis patients and controls

This study included 97 RA patients: 85 (87.6%) females and 12 (12.4%) males (7.1:1) with a mean age of 47.9 ± 8.9 years (35–70 years). The 45 controls were age (45.9 ± 8.1 years) and sex (39 females and 6 males; F:M 6.5:1) matched (*p* = 0.18 and *p* = 0.88, respectively). Characteristics, osteoporotic risk factors, DEXA, TBS, and FRAX of patients and controls are presented in Table 1 and Fig. 1. 86.6% of patients vs 31.1% control were not working (*p* < 0.0001) while 76.3% were married vs 91.1% (*p* = 0.01). Twenty-eight patients had their faces only exposed to the sun while 7 patients and 3 controls had their faces, arms, and legs exposed. The mean tender joint count (TJC) in the patients was 7.6 ± 6.9 and swollen joint count (SJC) was 1.6 ± 2.2. According to the DAS28, 61 (62.9%) had moderate, 27 (27.8%) high, and 6 (6.2%) low activity, while 3 (3.1%) were in remission.

**Table 1:** Characteristics, osteoporotic risk factors, dual-energy X-ray absorptiometry (DEXA), trabecular bone score (TBS), fracture risk assessment (FRAX) in 10 years, and vitamin D receptor (VDR) genotype in rheumatoid arthritis patients and controls

Variables, mean ± SD or *n* (%)	RA cases (*n* = 97)		Control (*n* = 45)		*p*
Age (years)	47.9	± 8.9	45.9	± 8.1	0.18
*Sex*					
Female:male	88:12		39:6		0.88
Disease duration (years)	9.4 ± 6.2 (1–25)		-		**-**
Age at onset (years)	38.4 ± 9 (20–62)		-		**-**
Smoking	5	(5.2)	3	(6.7)	0.73
BMI	26.4	± 3.5	27.1	± 3.5	0.24
Menopausal	34	(35.1)	9	(20)	**0.049**
Menopause age (years)	46.3	± 3.6	47.4	± 1.7	0.17
Diet rich in calcium	21	(21.6)	6	(13.3)	0.21
Sun exposure	35	(36.1)	3	(6.7)	** < 0.0001**
Prior fracture	10		0 (0)		-
Co-morbidities	17 (17.5)		0 (0)		-
Metabolic syndrome	6 (6.2)		0 (0)		-
MHAQ	0.6 ± 0.4 (0.1–1.8)		-		-
DAS28	4.52 ± 1.04		-		-
DEXA					
*Spine*					
T-score	− 0.99 ± 1.46		1.8 ± 0.27		** < 0.0001**
Osteopenia	38 (39.2)		4 (8.9)		**0.008**
Osteoporosis	9 (9.3)		4 (8.9)		
*Femur*					
T-score	− 0.46 ± 1.18		1.06 ± 1.44		** < 0.0001**
Osteopenia	24 (24.7)		6 (13.3)		0.051
Osteoporosis	1 (1)		0 (0)		
*Forearm*					
*T*-score	− 2.36 ± 1.22		− 0.61 ± 1.28		** < 0.0001**
Osteopenia	47 (48.5)		12 (26.7)		** < 0.0001**
Osteoporosis	43 (44.3)		1 (2.2)		
TBS	1.49 ± 0.16		1.46 ± 0.15		0.29
*Degradation*					
Partial	15 (15.5)		9 (20)		0.57
Severe	2 (2.1)		1 (2.2)		
FRAX					
Major OP #	4.65 ± 2.36		2.42 ± 0.95		** < 0.0001**
Hip #	0.73 ± 0.75		0.17 ± 0.33		** < 0.0001**
VDR genotype					
rs2228570 (FokI)					
GG	59 (60.8)		21 (46.7)		0.12
AG	33 (34)		20 (44.4)		
AA	5 (5.2)		4 (8.9)		
rs731236 (TaqI)					
AG	97 (100)		45 (100)		**-**

Bold values are significant at *p* < 0.05

*RA*, rheumatoid arthritis; *BMI*, body mass index; *TJC*, tender joint count; *SJC*, swollen joint count; *DAS28*, disease activity score; *MHAQ*, modified health assessment questionnaire; *DEXA*, dual energy x-ray absorptiometry; *TBS*, trabecular bone score; *FRAX*, fracture risk assessment in 10 years; *OP*, osteoporosis; *#*, fracture; *VDR*, vitamin D receptor

The laboratory investigations and mediations received by the patients are presented in Table 2. The vitamin D level was comparable with the control (9.3 ± 6.5 vs 10.4 ± 7.5 ng/mL, *p* = 0.4). None of the patients was receiving anti-osteoporotic therapy or biologic therapy.

**Table 2:** Laboratory investigations among rheumatoid arthritis cases

Mean ± SD (range) or *n* (%)	RA patients (*n* = 97)		
*Laboratory investigations*			
Hemoglobin (g/dL)	11.6 ± 1.2		Anemia: 48 (49.5)
TLC (× 10^3^/mm^3^)	6.5 ± 1.8		Leukopenia: 4 (4.1)
Platelets (× 10^3^/mm^3^)	288.1 ± 64.7		Thrombocytopenia: 6 (6.2)
ESR (mm/h)	46.1 ± 21.1		↑ESR: 76 (78.4)
CRP (mg/L)	33.1 ± 17.7		Positive CRP: 51 (52.6)
ALT (IU/L)	22.1 ± 10.9		↑LFT: 12 (12.4)
AST (IU/L)	24.8 ± 11.4		
Creatinine (mg/dL)	0.81 ± 0.56		Normal in all
Serum uric acid (mg/dL)	3.8 ± 0.95		Normal in all
Cholesterol (mg/dL)	145.9 ± 33.4		Dyslipidemia: 10 (10.3)
Triglycerides (mg/dL)	90.6 ± 38.7		
LDL (mg/dL)	79.3 ± 14.2		
HDL (mg/dL)	66.3 ± 13.6		
TSH (mIU/L)	1.42 ± 1.4		↑: 2 (2.1)/↓:14 (14.3)
PTH (pg/mL)	41.3 ± 31.6		↑: 5 (5.2)/↓:2 (2.1)
Rheumatoid factor (IU/mL)	37.1 ± 18.5		Positive: 71 (73.2)
Anti CCP (AU/mL)	132.9 ± 118.9		Positive: 75 (77.3)
Calcium			
Total (mg/dL)	9.3 ± 0.62		↓: 18 (18.6)
Ionized (mg/dL)	4.6 ± 0.62		↓: 16 (16.5)
ALP (U/L)	75.9 ± 40.3		↑ALP: 8 (8.2)
Vitamin D (ng/mL)	9. 3 ± 6.5 (2.2–33)		*Def.* 69 (71.1)/*insuff.*: 27 (27.8)
*Medications/Supplements*			
Methotrexate	11	(11.3)	
Leflunomide	94	(97)	
Hydroxychloroquine	90	(92.8)	
Steroids	20	(20.6)	Cumm. dose (mg): 72.7 ± 64.1
Calcium	46	(47.4)	
Vitamin D	6	(6.2)	

*RA*, rheumatoid arthritis; *TLC*, total leukocytic count; *ESR*, erythrocyte sedimentation rate; *CRP*, C-reactive protein; *ALT*, alanine transferase; *AST*, aspartate transaminase; *TSH*, thyroid stimulating hormone; *PTH*, parathyroid stimulating hormone; *Anti-CCP*, anti-cyclic citrullinated peptide; *ALP*, alkaline phosphatase

A comparison of the characteristics of the RA patients according to the BMD is presented in Table 3. On comparing the various parameters according to the VDR genotyping, the DEXA T-scores, TBS, and FRAX were similar. All the osteoporotic risk factors, DAS28, MHAQ, vitamin D level, RF, and anti-CCP were comparable. Only the ESR and CRP were significantly higher in those with rs2228570 AA genotype (73.4 ± 17.4 mm/1 h and 63.3 ± 22.7 mg/dL) compared to those with GG and AG (39.5 ± 16.7 mm/1 h, 25.5 ± 9.6 mg/dL and 47.4 ± 21.7 mm/1 h, 33.9 ± 16.9 mg/dL; *p* = 0.002 and *p* < 0.0001).

**Table 3:** Characteristics of the rheumatoid arthritis patients according to the bone mineral density

Variables, mean ± SD or *n* (%)	BMD in RA patients (*n* = 97)			*p*
	Normal (*n* = 6)	Osteopenia (*n* = 48)	OP (*n* = 43)	
Age (years)	38.2 ± 2.4	45.8 ± 6.6	51.7 ± 10	** < 0.0001**
Disease duration (years)	4.2 ± 2.8	7.5 ± 5.4	12.3 ± 6.3	** < 0.0001**
BMI	26.7 ± 1.5	26.6 ± 3.8	26.1 ± 3.3	0.76
Smoking	0 (0)	4 (8.3)	1 (2.3)	0.37
Passive smoking	2 (33.3)	14 (29.2)	16 (37.2)	0.72
Menopausal	0 (0)	13 (27.1)	21 (48.8)	**0.005**
Diet rich in calcium	3 (50)	8 (16.7)	10 (23.3)	0.17
Sun exposure	2 (33.3)	12 (25)	21 (48.8)	0.06
ESR (mm/L h)	39.2 ± 15.3	46.8 ± 21.5	46.2 ± 21.6	0.71
CRP (mg/dL)	3 (50)	25 (52.1)	23 (53.5)	0.98
RF positive	1 (16.7)	36 (75)	34 (79.1)	**0.004**
Anti-CCP positive	3 (50)	35 (72.9)	37 (86)	0.09
TSH (mIU/L)	0.98 ± 0.8	1.4 ± 1.5	1.5 ± 1.4	0.72
PTH (pg/mL)	38.3 ± 9.6	40.1 ± 20.6	43.1 ± 42.3	0.88
Vitamin D (ng/mL)	13.5 ± 9.2	8.9 ± 6	9.3 ± 6.5	0.26
DAS-28	4.6 ± 0.59	4.6 ± 0.88	4.4 ± 1.3	0.79
MHAQ	0.25 ± 0.19	0.58 ± 0.4	0.62 ± 0.4	0.11
Steroids	0 (0)	11 (22.9)	9 (20.9)	0.43
Methotrexate	0 (0)	5 (10.4)	6 (14)	0.59
Leflunomide	6 (0)	47 (97.9)	41 (95.3)	0.71
Hydroxychloroquine	6 (100)	45 (83.8)	39 (90.7)	0.8

Bold values are significant at *p* < 0.05

*BMD*, bone mineral density; *RA*, rheumatoid arthritis; *BMI*, body mass index; *ESR*, erythrocyte sedimentation rate; *CRP*, C-reactive protein; *RF*, rheumatoid factor; *Anti-CCP*, anti-cyclic citrullinated peptide; *TSH*, thyroid stimulating hormone; *PTH*, parathyroid stimulating hormone; *DAS28*, disease activity score; *MHAQ*, modified health assessment questionnaire

The TBS was significantly lower and FRAX (major and hip) higher in patients with positive RF (1.4 ± 0.2, 4.9 ± 2.5, and 0.8 ± 0.8) compared to those with negative (1.5 ± 0.1, 3.9 ± 1.9, and 0.4 ± 0.6; *p* = 0.001, *p* = 0.026, and *p* = 0.008). Those with positive anti-CCP had a significantly lower BMD (spine, femur, and forearm) and TBS and higher FRAX (− 1.21.3, − 0.7 ± 1.2, − 2.5 ± 1.2, 1.4 ± 0.2, 4.9 ± 2.5, and 0.8 ± 0.8) compared to those negative (− 0.2 ± 1.7, 0.3 ± 0.8, − 1.8 ± 1.3, 1.5 ± 0.1, 3.7 ± 1.5, and 0.4 ± 0.5; *p* = 0.02, *p* < 0.0001, *p* = 0.027, *p* = 0.002, *p* = 0.005, and *p* = 0.001).The FRAX for major OP # was significantly higher in those receiving steroids (5.6 ± 2.2) compared to those not (4.4 ± 2.4; *p* = 0.045); the femur *T*-score was significantly lower in those on LFN (− 0.5 ± 1.2) compared to those not (0.4 ± 0.2; *p* = 0.001) and the *T*-score spine was lower in those receiving HCQ (− 0.9 ± 1.5) compared to those not (− 1.7 ± 0.5; *p* = 0.006).

**Fig. 2:**
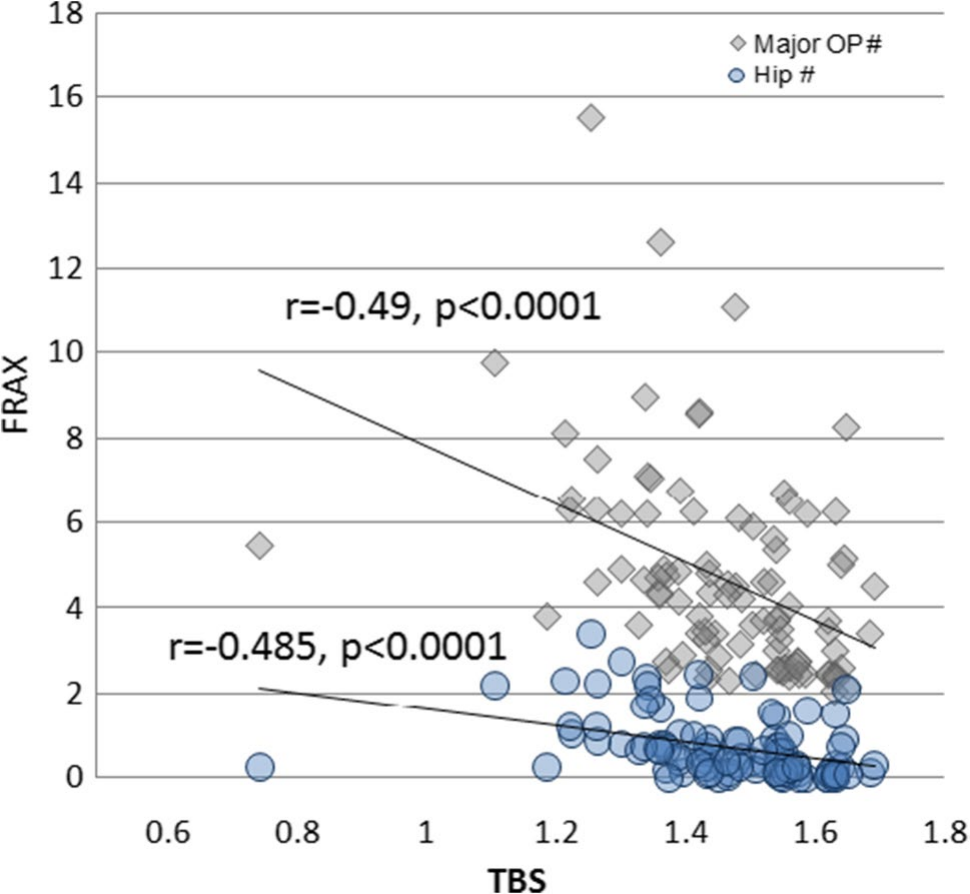
Correlation of the trabecular bone score (TBS) with the FRAX (major osteoporotic fracture and hip fracture) in rheumatoid arthritis patients

Correlations of the patients’ and disease characteristics with the TBS and FRAX in RA patients are shown in Table 4. The TBS in patients significantly correlated with the FRAX (*r* =  − 0.49, *p* < 0.0001) (Fig. 2).

**Table 4:** Correlations of the patients’ and disease characteristics with the trabecular bone score (TBS) and fracture risk assessment (FRAX) in 10 years in rheumatoid arthritis patients

Variable, *r* (*p*)	RA patients (*n* = 97)		
	TBS	FRAX	
		Major OP #	Hip #
Age (years)	− 0.55 **(< 0.0001)**	0.69 **(< 0.0001)**	0.74 **(< 0.0001)**
Disease duration	− 0.27 **(0.007)**	0.3 **(0.003)**	0.31 **(0.002)**
BMI	0.04 (0.73)	− 0.15 (0.15)	− 0.29 **(0.004)**
ESR (mm/1 h)	− 0.16 (0.13)	0.13 (0.22)	0.13 (0.22)
CRP (mg/dL)	− 0.12 (0.39)	− 0.01 (0.96)	0.06 (0.7)
Hemoglobin (g/dL)	0.13 (0.22)	− 0.15 (0.15)	− 0.16 (0.13)
TLC (× 10^3^/mm^3^)	0.09 (0.4)	0.04 (0.67)	− 0.1 (0.35)
Platelet (× 10^3^/mm^3^)	− 0.11 (0.27)	0.1 (0.32)	− 0.01 (0.91)
Creatinine (mg/dL)	− 0.05 (0.65)	0.09 (0.41)	0.15 (0.15)
SUA (mg/dL)	− 0.32 **(0.001)**	0.23 **(0.03)**	0.21 **(0.04)**
LDL (mg/dL)	− 0.1 (0.31)	0.22 **(0.03)**	0.21 **(0.036)**
Calcium			
*Total*	0.04 (0.67)	0.02 (0.83)	− 0.03 (0.75)
*Ionized*	0.05 (0.64)	− 0.12 (0.24)	− 0.14 (0.18)
ALP (U/L)	− 0.27 **(0.008)**	0.23 **(0.02)**	0.32 **(0.002)**
RF (IU/mL)	0.05 (0.68)	− 0.05 (0.7)	− 0.12 (0.3)
Anti-CCP (AU/mL)	− 0.3 **(0.008)**	0.15 (0.2)	0.13 (0.26)
TSH (mIU/L)	0.11 (0.29)	0.12 (0.25)	0.14 (0.18)
PTH (pg/mL)	− 0.2 (0.055)	− 0.02 (0.88)	− 0.08 (0.47)
Vitamin D (ng/mL)	0.06 (0.53)	− 0.08 (0.42)	0.07 (0.47)
DAS-28	− 0.16 (0.12)	0.06 (0.55)	0.09 (0.4)
MHAQ	− 0.49 **(< 0.0001)**	0.36 **(< 0.0001)**	0.32 **(< 0.0001)**

Bold values are significant at *p* < 0.05

*RA*, rheumatoid arthritis; *TBS*, trabecular bone score; *FRAX*, fracture risk assessment in 10 years; *OP*, osteoporosis; *#*, fracture; *ESR*, erythrocyte sedimentation rate; *CRP*, C-reactive protein; *TLC*, total leukocytic count; *SUA*, serum uric acid; *LDL*, low density lipoprotein; *ALP*, alkaline phosphatase; *RF*, rheumatoid factor; *Anti-CCP*, anticitrullinated cyclic peptide; *TSH*, thyroid stimulating hormone; *PTH*, parathyroid stimulating hormone; *DAS28*, disease activity score; *MHAQ*, modified health assessment questionnaire

There was a significant association between considering a diet rich in calcium (*β* 0.5, 95% CI 0.01–0.99; *p* = 0.046), the presence of metabolic syndrome (*β* − 1.45, 95% CI − 2.58 to − 0.31; *p* = 0.015), and dyslipidemia (*β* 1.18, 95% CI 0.26–2.1; *p* = 0.014) with the risk of osteoporosis in RA. The VDR rs2228570 genotyping (FokI) could not predict RA disease from control (AUC 0.43, 95% CI 0.32–0.53; *p* = 0.15) or patients with and without osteoporosis (AUC 0.48, 95% CI 0.37–0.6; *p* = 0.8). Only the age at onset and receiving MTX would significantly predict the TBS in RA patients (AUC 0.91, 95% CI 0.85–0.98; *p* = 0.04 and AUC 0.95, 95% CI 0.89–1; *p* = 0.03).

## Discussion

The coexistence of RA and osteoporosis is common and can lead to serious complications. The role of vitamin D and vitamin D receptor (VDR) gene polymorphisms in RA pathogenesis and clinical course of the disease has been investigated, with controversial data according to many factors including genetic, ethnicity, and environmental factors [19].

The current study showed that there was a significant difference between patients and controls as regards DXA, TBS, and FRAX. A lower BMD in RA patients compared to controls has been shown with an increased prevalence of osteoporosis [20]. The effectiveness of TBS for assessing the fracture risk in RA has been evaluated [21]. This was confirmed by Choi et al. [22] who revealed that vertebral fractures can be observed in the first year of the disease and one-third report a fracture within 5 years of follow-up. Syngle and colleagues showed that active RA patients have increased FRAX score, even in the absence of BMD findings indicating increased 10-years probability of major osteoporotic fracture and hip fracture [23]. A meta-analysis of 13 studies showed a significant higher risk of bone fracture in patients with RA with a higher frequency of vertebral and hip fracture [24]. However, in another study, the FRAX estimated with and without BMD may vary substantially in RA patients [25]. It should be considered that FRAX has some limitations. It does not consider, for example, the number of previous fractures, doses of glucocorticoids, and smoking or alcohol and does not include lumbar spine BMD or the number of falls [26].

There was no significant difference between cases and controls as regards vitamin D level as most of them had deficiency. Similarly, other studies demonstrated that there was no significant difference in serum vitamin D level in RA and controls [27, 28]. Vitamin D deficiency was present in almost all Egyptian healthy adolescents [29] and prevalent among Egyptian women of childbearing age [30]. There was a significant association between lack of physical activity, sun exposure, and vitamin D deficiency. Although Egypt is a sunny country, such vitamin D deficiency could be attributed to the special pattern of conservative clothing and the lack of outdoor physical activity in females. Vitamin D supplementation seems to be mandatory to halt the problem [29]. In contrast, others showed significant inverse correlation between vitamin D and disease onset in RA [31, 32]. Elbassiony et al. [33] have detected that the serum vitamin D in RA patients was significantly lower than that of controls.

A series of polymorphisms in VDR gene have been reported. They include BsmI, ApaI, TaqI restriction sites, variable PolyA length, and FokI restriction site. Of these polymorphic sites, BsmI are substitutions on intron 8 whereas ForkI restriction enzyme identifies a polymorphic site in exon 2 at the 50 ends of the VDR gene. They have an effective role, a functional significance of these polymorphisms, and their potential effects on RA susceptibility [34]. The current study showed that VDR genotyping rs 2,228,570 (Fok-I) was similar between patients and control with a tendency to a higher frequency of GG genotype. There was no significant difference as regards rs731236 (Taq-I) genotyping between patients and controls. In harmony, genetic analysis of four VDR polymorphisms, BsmI, FokI, ApaI, and TaqI did not confer the susceptibility to RA in Lithuanian population [19]. However, it has been reported that there was a significant higher percentage of (Fok-I) genotyping among RA cases versus controls but not BsmI [35]. The frequencies CGAT, CGGA, CGGT, CTAA, CTAT, TGAA, TGAT, TGGA, and TTGA haplotypes were higher in patients and act as risk factors of RA onset [28]. A recent study revealed that VDR Fok1 (rs10735810) polymorphism had an association with RA, while VDR Bsm1 (rs1544410) polymorphism has not [36].

It was detected in the current study that there was a significant difference relation of VDR genotype (Fok-I) and CRP levels among cases but not with other parameters. Virtually, all immune cells express VDR, making them susceptible to vitamin D-mediated modulation, so after binding to VDR, active vitamin D has a direct immunosuppressive effect on dendritic cells, reduces CD4 + cells proliferation, and differentiation into Th1 and Th17. VDR agonists were proposed to be selective inhibitors of Th1 cell development and found to inhibit Th1-type cytokines such as IL-2 and TNF-α directly [37]. Furthermore, a study found that variants of FokI Ff genotype were associated with a higher CRP concentration in RA subjects [19].

There was a significant association between the presence of osteoporosis and age, disease duration, presence of menopause, and RF positivity. The TBS was significantly lower and FRAX higher in patients with positive RF. Those with positive anti-CCP had a significantly lower BMD and TBS as well as a higher FRAX. The FRAX for major OP # was significantly higher in those receiving steroids; the femur *T*-score was significantly lower in those on LFN and the *T*-score spine was lower in those receiving HCQ. The FRAX was significantly related and the TBS inversely with the age, disease duration, SUA, ALP, and MHAQ. The FRAX was further significantly related to the LDL level and the TBS negatively with the anti-CCP titre. In concordance, Haugeberg et al. showed that reduced BMD was associated with age, current use of corticosteroids, physical disability, and RF positivity in RA [20]. Lee and colleagues reported that postmenopausal patients with RA 46.8% were osteoporotic with a higher frequency of previous fractures, especially of the femur and wrist. They further added that advanced age, longer disease duration, higher cumulative dose of steroids, and higher HAQ score were significant key players associated with osteoporosis [38]. Choi et al. [39] revealed that osteoporotic fractures were detected in 16.9% of postmenopausal patients; female gender, age, disease duration, and steroids dose were independent risk factors for fracture. In Tunisian RA patients, bone loss was significantly associated with age, longer disease duration, RF, atlantoaxial subluxation, and corticosteroids use. The risk of multiple osteoporotic and hip fracture was associated with age, menopause, calcium intake, disease duration, HAQ, cumulative dose, and duration of corticosteroids [40].

Kwok and others concluded that all men (aged ≥ 70 years) and women (aged ≥ 65 years) should receive universal DEXA assessment for osteoporosis and those with an increased FRAX should receive anti-osteoporotic treatment to prevent hip fractures [41].

Among the study limitations is the relatively small sample size and assessing only two VDR genes for their association with the risk of bone loss. So, further multi-center research is necessary among larger cohorts, involving different geographical distribution.

In conclusion, the reduced BMD and increased tendency to fractures are remarkable in RA patients. Vitamin D level was decreased in patients and control, and the VDR gene polymorphism Fok-I and Taq-1 were not linked to RA. TBS and FRAX are effective tools to assess osteoporotic fractures in RA. Bone loss and fragility fracture are related to age, disease duration, menopause, SUA, RF and anti-CCP positivity, function impairment, and steroids.
